# Recycling of Polyurethanes via Covalent Adaptable Networks: The Role of Crosslink Density in Performance Recovery

**DOI:** 10.3390/polym17202778

**Published:** 2025-10-17

**Authors:** Edoardo Miravalle, Teodora Andra Olariu, Claudio Cecone, Valentina Brunella, Pierangiola Bracco, Marco Zanetti

**Affiliations:** 1 Department of Chemistry, NIS Interdepartmental Centre, University of Turin, Via Pietro Giuria 7, 10125 Turin, Italy; edoardo.miravalle@unito.it (E.M.); teodora.olariu@edu.unito.it (T.A.O.); claudio.cecone@unito.it (C.C.); valentina.brunella@unito.it (V.B.); pierangiola.bracco@unito.it (P.B.); 2 Instm Reference Centre, University of Turin, Via G. Quarello 15A, 10135 Turin, Italy; 3 SUSPLAS@UniTo, Sustainable Plastic Scientific Hub, University of Turin, Via Pietro Giuria 7, 10125 Turin, Italy

**Keywords:** recycling, crosslink density, CAN, DCC, thermoset, polyurethane

## Abstract

Thermoset polyurethanes invite industrial interest for their versatility and chemical and mechanical resistance due to their permanently crosslinked networks; yet this structural feature severely limits their recyclability. Recent advances in Covalent Adaptable Networks (CANs), enabled by Dynamic Covalent Chemistry (DCC), have demonstrated promising pathways toward reprocessability through bond-exchange mechanisms. However, no clear link has yet been identified between material properties and the retention of performance after reprocessing. This work investigates the role of crosslink density as a key factor in determining the reprocessability of polyurethane networks. Two model systems with comparable compositions but distinct crosslink densities were synthesised, reprocessed, and compared. Relaxation analysis based on the Maxwellian approach proved insufficient to predict reprocessing outcomes. Only the highly crosslinked network yielded homogeneous reprocessable films with significant retention of mechanical performance, whereas the less crosslinked network resulted in incoherent materials with markedly reduced properties. The application of Kohlrausch–Williams–Watts (KWW) fitting revealed that dynamic covalent exchange dominates relaxation in the highly crosslinked system, while in the looser network, relaxation is governed by soft segment mobility, hindering effective network reformation. These findings underscore the pivotal role of crosslink density in determining the recyclability of thermoset polyurethanes and provide new insights for the rational design of reprocessable materials.

## 1. Introduction

Thermosets, together with thermoplastics, represent the two principal classes of polymeric materials. They are defined by a three-dimensional, permanently crosslinked chemical network, which imparts superior chemical resistance and mechanical performance compared with their thermoplastic counterparts. However, the very feature that makes these materials attractive also constitutes a major drawback: the fixed network architecture severely limits the application of conventional recycling strategies, such as mechanical reprocessing [1].

In recent years, extensive research on CANs has demonstrated that the incorporation of bonds following the principles of DCC can provide a viable means to address this limitation. CANs can be activated to enable functions such as stimuli-responsiveness [2], self-healing [3], or recyclability [4]. Various dynamic covalent bonds can be exploited for this purpose, which generally operate through two main exchange mechanisms, associative or dissociative, the former of which is governed by kinetics and the latter is governed by thermodynamics, and each one has specific features [5,6].

Polyurethanes have attracted particular attention due to their large-scale production and widespread use, which make them the most extensively manufactured thermoset polymers worldwide [7]. Owing to the wide variety of building blocks available for their synthesis, polyurethanes can give rise to networks with highly diverse structures. Consequently, the application of DCC must be evaluated on a case-by-case basis, since exchange mechanisms may contribute in different ways depending on the specific network architecture [8,9]. Differences in composition also resulted in markedly different outcomes during the reprocessing experiments. In some cases, the reprocessed materials exhibited reduced mechanical performance [10,11,12,13], whereas for other properties, retention was observed [14,15,16,17], and in certain instances, even enhanced properties were reported [18,19,20,21]. To the best of our knowledge, no comprehensive explanation has yet been provided to establish a clear correlation between the retention, or improvement, of mechanical and thermal properties and the intrinsic characteristics of the material.

Within the context of plastic recycling, it is crucial to understand how specific material parameters influence the reprocessing process, and which of these can be effectively adjusted to obtain a reprocessed material with properties comparable to the pristine polymer. Among the most significant parameters in thermosets is crosslink density, which has recently been investigated regarding its role in reprocessing strategies [22,23] and its impact on the final material properties [24]. We propose that this parameter is also a key factor governing the extent to which mechanical properties are retained following reprocessing.

To specifically evaluate the role of crosslink density on the recyclability of thermoset polyurethanes, we deliberately restricted our experimental design to two model systems with comparable chemistry but markedly different crosslink densities. By keeping the chemical composition constant and varying only the average molecular weight and functionality of the polyol precursors, we were able to isolate the effect of crosslink density from other structural factors. This targeted approach was chosen to unambiguously establish the correlation between crosslink density and the retention of mechanical properties after reprocessing.

In this work, we present a study on the role of crosslink density and its influence on the retention of mechanical properties. Two polyurethane networks with comparable compositions but differing crosslink densities were synthesised and characterised using FTIR, TGA, DSC, DMA, and tensile testing. Relaxation behaviour was initially analysed through the classical Maxwellian approach to determine τ* and the corresponding reprocessing conditions; however, the results proved unsatisfactory. A homogeneous material could be reprocessed only in the case of the more highly crosslinked network, which also displayed good retention of mechanical performance. By contrast, the less densely crosslinked system yielded a less continuous film with significantly reduced properties.

To further elucidate these observations, a Kohlrausch–Williams–Watts (KWW) fitting was applied. The ability to achieve an effective reprocessed material was attributed to the more efficient dynamic covalent chemistry operating in the highly crosslinked network, whereas in the less crosslinked system, competing relaxation processes interfered with bond reformation, thereby preventing the recovery of a coherent network structure. We suggest that this approach offers a useful contribution towards narrowing the knowledge gap in the reprocessing of thermoset polyurethanes.

## 2. Materials and Methods

### 2.1. Materials

**Figure 1:**
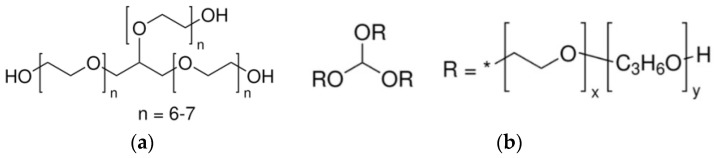
Structure of polyols used (**a**) glycerol ethoxylate and (**b**) glycerol ethoxylate-co-propoxylate.

For the synthesis of the networks, two different polyols were used, glycerol ethoxylate (M_n_ ~ 950 g/mol) and glycerol ethoxylate-co-propoxylate (M_n_ ~ 5300 g/mol), both are polyether polyols with a functionality of 2.5–3, structure reported in Figure 1, toluene diisocyanate (TDI) (MM = 172.2 g/mol), glycerol and dibutyltin dilaurate (DBTDL) as catalyst. For the swelling test, dimethylformamide (DMF) (≥99%, CAS 68-12-2) was used. All reagents were purchased from Sigma-Aldrich (St. Louis, MO, USA).

### 2.2. Synthesis of Polyurethane Networks

During the synthesis, two different networks (PU1 and PU2) were created based on the polyol system used. PU1 was synthesised with ethoxylated glycerol, PU2 with a mixture of ethoxylated-co-propoxylated glycerol and glycerol (1:1 OH molar ratio). Both polyol moieties were pre-treated at 80 °C under vacuum for 4 h to remove residual moisture. The synthesis followed the same steps for each formulation.

Initially, each polyol moiety was mixed with DBTL (0.5% *w*/*w* with respect to the weight of polyol used), and the mixture was then degassed at room temperature. Subsequently, the polyol mixture was combined with TDI in a stoichiometric ratio (hydroxyl: isocyanate groups = 1:1). Once mixed, the formulations were placed into moulds and cured overnight at 70 °C.

### 2.3. Reprocessing Conditions

The proposed reprocessing method is mechanical and consists of two phases. The first phase involves shredding the material using an ultra-centrifugal rotor mill, Retsch ZM 300 (Haan, Germany), equipped with a 1 mm sieve. Before reprocessing, the samples were immersed in liquid nitrogen to facilitate fragmentation and prevent system degradation due to the heat generated during milling. In the second phase, the resulting powder was subjected to hot pressing. The temperature used was identical for both materials, 170 °C, while the processing times were 60 min and 15 min (these values were determined by DMA analysis, as reported later). A pressure of 60 MPa was applied to all samples.

### 2.4. Characterisation

FTIR spectroscopy was used to evaluate the chemical structures of the pristine and the reprocessed samples. Measurements were carried out with a Spectrum 100 (Perkin Elmer, Buckinghamshire, UK) via the ATR technique equipped with a germanium crystal. The spectra were measured at room temperature in the wavelength range of 650–4000 cm^−1^ with a resolution of 4 cm^−1^, acquiring 8 scans. Additionally, spectra were obtained in a temperature ramp to gain insight into the exchange mechanism. Measurements were performed using a Spectrum Spotlight 300 FT-IR microscope (Perkin Elmer, Buckinghamshire, UK). Samples were deposited onto a metallic reflective support, and spectra were collected in reflection/absorption mode. Controlled and precise heating of the sample was achieved by integrating the microscope with a modified Linkam THMS600 heating stage (Linkam, Salfords, Redhill, UK), operated via a Linkam TMS94 stage temperature controller (Linkam, Salfords, Redhill, UK). The spectra were obtained at incremental temperatures between room temperature and 200 °C, with 10 °C intervals and a 2 min isothermal period. The wavelength range was set to 650–4000 cm^−1^, with a resolution of 2 cm^−1^.

TGA analysis of all samples was performed using a TGA Q500 (TA instruments, New Castle, DE, USA) within the temperature range of 40–800 °C, with a ramp rate of 10 °C/min in a N_2_ atmosphere until 700 °C, then switching to air up to 800 °C at a flow rate of 60 mL/min on the sample. The mass of all samples was approximately 12 mg.

DSC was used to evaluate the T_g_ of the specimens. Analysis was performed with a DSC Q200 (TA Instruments, New Castle, DE, USA). The temperature varies between −90 °C and 120 °C with a 10 °C/min ramp under 50 mL/min N_2_ flow. The analysis consisted of two heating and one cooling cycles, separated by an isothermal period of 5 min at the end of each cycle.

DMA analyses were carried out with a DMA 800Q Q800 (TA Instruments, New Castle, DE, USA) in tension mode. The samples were cut into rectangular shapes of 20 × 5 × 0.3 mm^3^. The tests were run between 40 °C and 190 °C using a 3 °C/min ramp and 1% strain at 1 Hz, with a 0.01 N preload force applied. The analyses were carried out in triplicate. The results allow to determine the storage modulus and the crosslink density (*υ_e_*) was determined according to the theory of rubber elasticity for small deformations on the rubbery plateau [25,26], applying Equation (1), being E^′^ the storage modulus in MPa, *R* the molar gas constant (8.314 J mol^−1^K^−1^), and T_0_ the fixed temperature suitable to compare the networks in Kelvin. The determination of *υ_e_* has been performed at *T*_0_ = 50 °C, as the temperature was selected within a range in which the module was not subject to fluctuations, thus remaining in the rubbery plateau.

(1) $$\upsilon_{e}=\frac{{E\prime }_{T_{0}}}{3RT_{0}},$$

Stress relaxation tests were conducted with the same DMA instrument using samples of the same dimensions and testing them at three different temperatures (150 °C, 160 °C and 170 °C). Once the desired temperature was reached, an isotherm of 5 min was applied before applying a constant deformation of 5% for 2700 s. The stress relaxation of a dynamic cross-linked network can be defined using the Maxwell model reported in Equation (2). According to this model and to the definition of relaxation time (τ*), the time required for the sample to reach the value of 1/e of the initial value of the elastic modulus [27]. Using the Arrhenius plot, Equation (3), the activation energy (E_a_) of the networks was determined.

(2) E(t)E0=e−tτ*,

(3) τ*=τ0eEaRT

Being *E* the relaxation modulus in MPa, *t* the time in seconds, τ_0_ the preexponential constant, *E_a_* the activation energy in KJ/mol, *R* the molar gas constant, and *T* the absolute temperature.

Relaxation data were further analysed through the application of the Kohlrausch–Williams–Watts (KWW) fitting, a stretched exponential decay model [28]. This model involves fitting the relaxation modulus data obtained via DMA using Equation (4) [28,29].

(4) $$\frac{E(t)}{E_{0}}=\frac{E_{perm}}{E_{0}}+\left(1-\frac{E_{perm}}{E_{0}}\right)\exp\left\{-{\left(\frac{t}{\tau^{KWW}}\right)}^{\beta }\right\},$$

where *E*(*t*)/*E*_0_ is the normalised relaxation modulus in a function of time *t*, *E_perm_*/*E*_0_ is the fraction of residual stress that remains in the network as time approaches infinity, *τ^KWW^* is the characteristic relaxation time, and β that controls the shape of the stretched exponential decay and reflects the breadth of the relaxation distribution, if equal to 1 the process involves a single relaxation time and the smaller the broader the distribution of relaxation times. The parameter *E_perm_*/*E*_0_ has been observed to be 0 for linear and branched polymers, as well as for CANs characterised by the absence of permanent bonds [28].

The fitting of the experimental data thus leads to the determination of the parameters *τ^KWW^* and *β*, which can be used to define the average relaxation time ⟨τ⟩, as reported in Equation (5).

(5) $$\langle \tau \rangle =\frac{\tau^{KWW}\Gamma \left(1/\beta \right)}{\beta },$$

where Γ is Euler’s Gamma function.

Uniaxial tensile measurements were conducted at room temperature on an Instron 3365, equipped with a 500 N load cell. The specimens were cut in dog-bone shape with dimensions according to ASTM D638 [30] type V. The tests were conducted at 5 mm/min displacement after applying a preload force of 0.06 N with a 2.5 mm/min displacement. For each sample, six specimens were tested.

The swelling measurements were performed in DMF [31]. PUs discs (5 mm diameter × 1 mm thickness) were placed in a closed vial with DMF (30 mL) at room temperature and in triplicate. The measurements were carried out after 24 h by removing the sample from DMF and quickly drying the excess solvent with a towel. The percentage of swelling (S%) was calculated using Equation (6):

(6) $$S\%=\frac{W-W_{0}}{W_{0}}\cdot 100,$$

*W*_0_ and *W* are the sample weights before and after swelling, respectively. The samples were then dried in a vacuum oven for 24 h at 120 °C and weighed to assess the loss in weight with Equation (7):

(7) $$\%Weight\;loss=100-(\frac{W_{D}}{W_{0}}\cdot 100),$$

where *W_D_* is the sample weights after drying.

## 3. Results and Discussion

### 3.1. Characterisation of Pristine Networks

**Figure 2:**
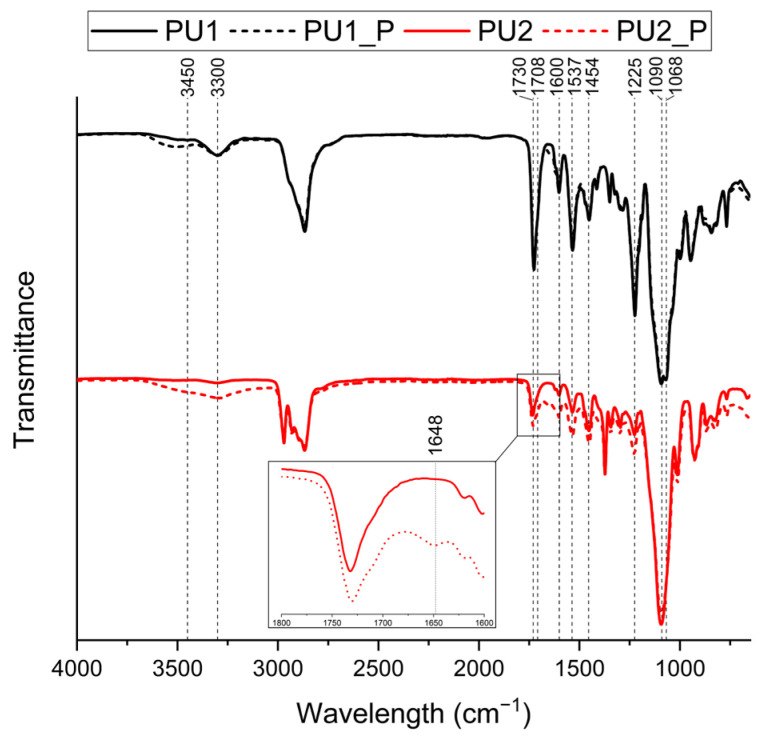
Comparison of FTIR-ATR spectra of the pristine and reprocessed sample.

Figure 2 displays the spectra of the two synthesised polyurethanes. In all cases, the characteristic peaks of polyurethanes are observed. The peak around 3300 cm^−1^ corresponds to the N–H stretching vibration, further confirmed by the peak at approximately 1537 cm^−1^, which corresponds to N–H bending. These N–H signals arise from the urethane linkages formed through the reaction of isocyanate (–NCO) groups with hydroxyl (–OH) groups during network synthesis. Each urethane bond contains a secondary amine moiety (–NH–), which is responsible for the stretching vibration at ~3300 cm^−1^ and the bending vibration at ~1537 cm^−1^, in agreement with the typical FTIR assignments reported for polyurethanes in the literature [32,33]. The urethane C=O bond is evidenced by peaks at 1730 cm^−1^ and 1225 cm^−1^, while the urethane C–N bond is identified at 1454 cm^−1^. Another relevant peak at 1600 cm^−1^ is attributed to the C=O bond of urea, formed as a side reaction during the formation of the urethane bond. Additional peaks associated with the polyol component appear around 3000 cm^−1^, corresponding to C–H bonds, and at 1090 cm^−1^, indicative of C–O–C stretching vibrations. Due to the similar composition, the spectra display a high degree of similarity; however, some differences can be observed.

Around 3300 cm^−1^, the presence of two overlapping peaks, one sharper and one broader, is particularly evident in PU1. At 3000 cm^−1^, different peaks can be observed for the two formulations. Additionally, PU1 exhibits in the carbonyl area a shoulder at 1708 cm^−1^, while in the ether area a peak at 1068 cm^−1^.

The differences in the C–H stretching region around 3000 cm^−1^ can be attributed to the type of polyol used, since PU1 contains only methylene C–H bonds, and PU2 also features methyl C–H bonds. However, the variations in the N–H, C=O, and C–O–C regions can be explained by the formation of hydrogen bonds between different polymer chains. Specifically, N–H hydrogens can interact with carbonyl or ether groups, generating signals corresponding to free or hydrogen-bonded species [32,33].

For free N–H, a signal appears at higher wavenumbers (approximately 3450 cm^−1^), while hydrogen-bonded N–H shifts to lower values (3300 cm^−1^). Similarly, free C=O groups absorb at 1730 cm^−1^, whereas hydrogen-bonded C=O shifts to 1708 cm^−1^. A similar shift is observed for the C–O–C bond, with free ether groups absorbing at 1090 cm^−1^ and hydrogen-bonded species at 1068 cm^−1^. These differences are more pronounced in PU1 than in PU2, likely due to the larger number of functional groups capable of forming such interactions.

Moreover, a higher intensity is observed for all peaks associated with urethane bonds as the molecular size of the polyol used in the network synthesis decreases. This confirms the synthesis conditions, where a higher amount of isocyanate was required for the same polyol weight as the polyol’s molecular weight decreased, further supporting the greater formation of urethane bonds. Additionally, no peaks corresponding to free isocyanate or polyol were observed, indicating that the reaction was completed.

**Figure 3:**
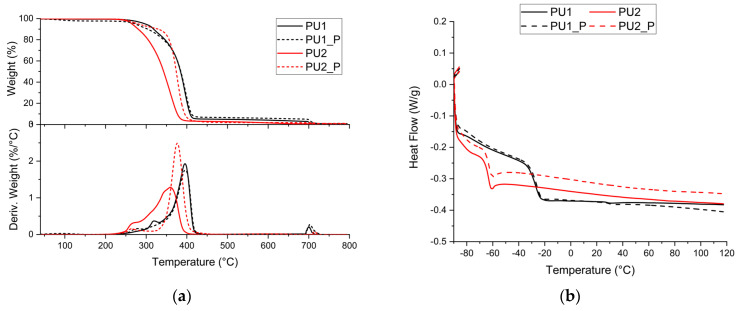
Overlaid thermal analysis of pristine and reprocessed materials (**a**) TGA and DTG, (**b**) DSC.

TGA analyses were performed to assess the thermal stability of the synthesised materials; the thermograms and their respective derivatives are reported in Figure 3a. It was observed that volatilisation of the systems begins at approximately 210 °C, a similar value for both synthesised materials, while the process ends at 410 °C and 436 °C for PU2 and PU1, respectively. Differences were also observed in the T_5%_ values, which were 265 °C for PU2 and 297 °C for PU1, as well as in T_max_, the temperature derived from the maximum of the first derivative, which was 360 °C and 396 °C for PU2 and PU1, respectively.

Both samples exhibited a similar degradation profile, characterised by two distinct degradation steps typical of polyurethanes: the first associated with urethane bond degradation and the second with the degradation of the polyol component [34,35]. 4 wt.% residue formation was observed before exposure to air for PU1. Given the structural similarity of the synthesised networks, similar thermograms were expected, particularly for the onset of degradation, because the same isocyanate was used. Additionally, a more complex second degradation step is observable in PU2 with respect to PU1.

According to the literature, major values of T_5%_ and T_max_ were observed in PU1, as materials with a higher crosslinking density exhibit greater thermal stability, as indicated by temperature values such as the onset of degradation and the maximum weight loss temperature [32,36].

Increased char formation further supports the successful synthesis of the desired networks, as a more densely crosslinked PU exhibits higher char yield than a less crosslinked one [35]. Additionally, a more pronounced first degradation step indicates greater degradation of the urethane bond within the system, which is more evident in PU1 than in PU2 [35]. Furthermore, the more complex second step related to the degradation of the soft component is justified by the use of a mixture of polyols that influences the degradation process [35,37].

DSC analyses were conducted to determine the T_g_ of the polyurethanes and are reported in Figure 3b. The obtained values were −27 °C and −63 °C for PU1 and PU2, respectively. These results indicate an increasing T_g_ with the use of trifunctional polyol of shorter chain length, in agreement with literature findings [38,39].

Swelling tests were performed in DMF to gain further insight into crosslink density; the data obtained are shown in Table 1. After the immersion and drying process, the swelling ratios observed were 196% and 242%, with weight loss values of 8% and 4% for PU1 and PU2, respectively.

The swelling values were statistically different, confirming that materials synthesised using higher molecular weight polyols exhibit lower crosslink density. This is expected, as greater swelling indicates a lower degree of crosslinking [39].

**Figure 4:**
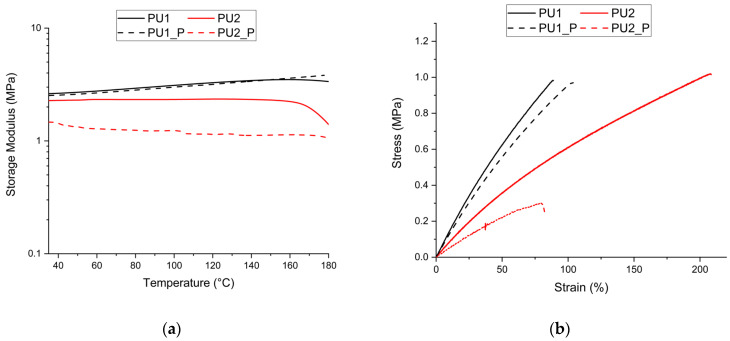
Comparison of properties obtained for pristine and reprocessed materials, (**a**) Storage modulus obtained with DMA; (**b**) stress–strain curves.

The storage modulus was determined as a function of temperature, since there is a correlation between this parameter and crosslink density, with representative curves reported in Figure 4a. For both materials, the initial part of the curves shows a similar trend, where they remain stable; however, their behaviour diverges as the temperature increases. PU1 exhibits a slight increase in modulus, with only a minor decrease occurring at 180 °C. On the contrary, PU2 shows a reduction in modulus at approximately 160 °C. An increase has already been observed for CAN systems and can be related to a proportional rubbery plateau to absolute temperature or temperature-driven stiffening of the network [22,40]. Meanwhile, regarding the decrease in modulus at high temperatures, since these temperatures are below the system’s thermal degradation threshold, it is believed that the modulus reductions are related to the system’s relaxation and the material’s loss of structural integrity. The phenomenon appears to be more significant in the material with lower crosslink density, suggesting that the process is linked to the material’s soft component and a more loosely connected network structure.

The storage modulus values at 50 °C were compared, as all samples exhibited a stable modulus at this temperature. The values obtained were 3.05 ± 0.47 MPa and 2.30 ± 0.27 MPa for PU1 and PU2, respectively. These values were used to calculate the crosslink density, resulting in 379 ± 58 mol/m^3^ for PU1 and 286 ± 33 mol/m^3^ for PU2.

DMA tests were conducted to assess the stress relaxation behaviour of the PU series and assess their dynamic properties and potential applicability within CAN theory. Each material was subjected to constant strain at a constant temperature while monitoring the normalised modulus trend E(t)/E_0_. This approach allows for the determination of a characteristic time, τ*, corresponding to the modulus value of 1/e of the initial modulus. This parameter helps to compare different PU samples and estimate the reprocessing time at a given temperature.

**Figure 5:**
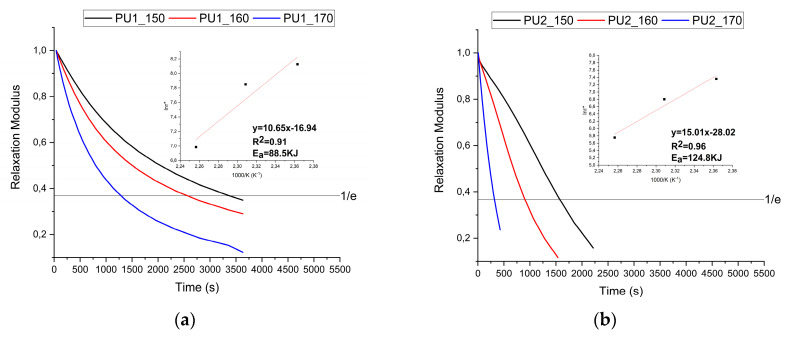
Stress relaxation analysis obtained at different temperatures with DMA for (**a**) PU1 and (**b**) PU2.

Each material was subjected to relaxation tests at three different temperatures: 150 °C, 160 °C, and 170 °C. At each tested temperature, the threshold value was achieved. Furthermore, as the temperature increases, the τ* value decreases, as shown in Figure 5a,b.

Specifically, observing the values obtained at 170 °C, there is a transition from 1100 s for PU1 to 315 s for PU2. This confirms the formation of different crosslinked materials when using polyols of higher molecular weight, as less crosslinked materials exhibit faster relaxation [41,42]. Through this analysis, the reprocessing conditions were also defined, being established at three times the τ* value.

Thus, both materials have been reprocessed at 170 °C, PU1 for 60 min, while PU2 for 15 min. These conditions were chosen to balance processing time and temperature, minimising the former while avoiding potential degradation phenomena.

Furthermore, obtaining τ* values at different temperatures made it possible to determine the activation energies of the various networks using an Arrhenius plot. The values obtained were 88.5 kJ and 124.8 kJ, PU1 and PU2.

Although this type of analysis and evaluation of network mobility is widely used and accepted for CANs, its limitations must be acknowledged. Specifically, it is essential to emphasise that the use of the τ* parameter was introduced in a study based on ideal networks, where it was assumed that a single event, covalent bond exchange, was solely responsible for material relaxation [43]. Applying this methodology to systems that deviate from those described in the original study could lead to misinterpretations, particularly considering the well-known challenges in accurately modelling polymer relaxation. Consequently, describing CAN relaxation using a single exponential decay model or a single relaxation time may not always be sufficient. The KWW fitting approach was applied to gain deeper insights into the relaxation behaviour of these systems by considering multiple contributions.

After the experimentally obtained DMA data were analysed, it was observed that the normalised modulus tended to have a sufficiently low value, allowing for the assumption of the absence of permanent bonds. Consequently, the preexponential contribution can be considered negligible [28].

Table 2 reports the optimised fitting parameters, including the characteristic relaxation time and β, along with the average relaxation values obtained. Comparing these with the previously determined τ* values, a substantial similarity with <τ> is observed. It can be observed that the values of <τ> and τ* are very similar, with a significant difference only for PU1 at 170 °C.

However, the most interesting parameter is β, which provides information on the number of relaxation processes that occur in the materials. PU2 shows unitary values, while PU1 exhibits values around 0.9. This suggests that PU2 undergoes a single relaxation phenomenon, while PU1 involves at least two processes. Although the model cannot provide specific information on the type of relaxation occurring, some speculation can be made. Given the structure of the materials, it is assumed that the relaxation processes involved in this type of system are primarily twofold. One is attributed to the relaxation of the linear component formed by the polyol segment. Meanwhile, the other is associated with relaxation driven by the exchange of covalent bonds at the network nodes, following CAN theory.

It is therefore presumed that in PU2, obtained with a higher molecular weight polyol, only one relaxation phenomenon occurs: the relaxation of the soft component, which can take place rapidly. In contrast, PU1 also exhibits a contribution from relaxation due to bond exchange, which requires higher temperatures and longer timescales.

An interesting observation is that the relaxation of both networks can, as a first approximation, be evaluated using the classical CAN method. However, due to the limited bond exchange processes in less densely crosslinked networks during relaxation, it is proposed that deriving reprocessing parameters from relaxation experiments might not truly represent the network’s reprocessability. Effective reprocessability requires both bond opening and network restructuring to maintain the original properties, highlighting the necessity for a more detailed approach in evaluating the recyclability of these networks.

Finally, the mechanical properties of the synthesised PU series were analysed, with representative stress–strain curves for each network reported in Figure 4b. The obtained tensile strength values were 0.93 ± 0.12 MPa and 0.96 ± 0.14 MPa for PU1 and PU2, respectively. Meanwhile, the elongation at break was 90 ± 16% for PU1 and 197 ± 35% for PU2.

Through these analyses of the pristine materials, it was possible to confirm the synthesis of PUs with different crosslinking densities by using polyols of varying molecular weights.

### 3.2. Reprocessing

**Figure 6:**
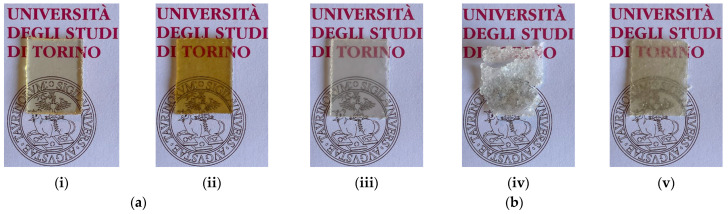
Reprocessing of PU networks (**a**) PU1 before (**i**) and after (**ii**) reprocessing with conditions retrieved via DMA; (**b**) PU2 before (**iii**) and after reprocessing, using reprocessing conditions retrieved via DMA (**iv**) and the same used for PU1 (**v**).

Once synthesised and characterised, the materials were successfully reprocessed using hot pressing. After grinding the materials to a uniform size of approximately 1 mm, 1–1.5 g of each sample was weighed and placed in a PTFE mould, spaced with a 0.2 mm aluminium mask. The reprocessed samples obtained were coded PU1_P and PU2_P, respectively, after reprocessing PU1 and PU2. The materials before and after reprocessing are reported in Figure 6. Interestingly, only for PU1 was it possible to reprocess the pristine material by applying the conditions obtained with DMA analysis. Meanwhile, for PU2, applying the reprocessing condition obtained through DMA led to a discontinuous film, and it was not possible to obtain an analysable artefact. Only by increasing the reprocessing time to match PU1 was it possible to obtain an analysable continuous film. For both reprocessed materials, a slight yellowing was observed. However, PU1_P maintained the transparency of the pristine material; meanwhile, PU2_P looks opaque with distinguishable constituent grains and matte texture.

**Figure 7:**
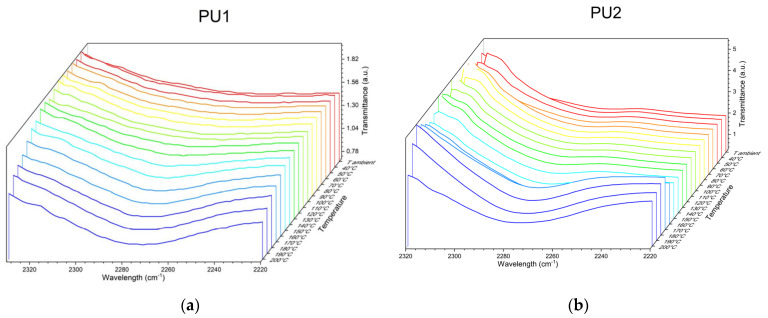
FTIR detail of NCO wavenumber for (**a**) PU1 and (**b**) PU2 collected at increasing temperatures from room temperature to 200 °C (10 °C intervals). The color gradient from blue to red indicates the temperature increase.

Temperature-ramped IR analyses were performed to gain further insight into the reprocessing mechanism. Figure 7a,b present a zoomed-in view of the spectra in the 2270 cm^−1^ region. These spectra show that as the temperature increases, a peak appears in this region and becomes more intense with rising temperature. This peak corresponds to a free isocyanate, which confirms that the reprocessing process in these materials follows a dissociative network-opening mechanism. Upon heating, the equilibrium between the urethane bond and its initial components, isocyanate and polyol, shifts in favour of the reactants. This results in a dynamic network that enables bond breakage and reformation, facilitating the creation of new artefacts.

Despite the structural differences among the networks, the peak appears at the same temperature, around 150 °C, and its intensity increases similarly.

Subsequently, the characterisation of the reprocessed materials was carried out using ATR-IR, TGA and DSC.

Figure 2 presents the IR spectra of the reprocessed materials compared to their pristine counterparts. For both reprocessed materials, no loss of the characteristic functionalities is observed. However, some discrepancies are visible for both materials. In the 3250–3750 cm^−1^ region, more prominently in PU2_P, a broad band overlapping the N–H stretching peak can be observed, indicating the generation of new hydroxyl groups, either as a consequence of oxidation during reprocessing or arising from urethane groups that underwent cleavage and failed to reform upon cooling. Additionally, PU2_P exhibits a new absorption in the carbonyl region centred at 1648 cm^−1^ attributed to the formation of biuret structures. The higher intensity of newly formed hydroxyl groups, together with the appearance of urethane-like species in PU2_P, is consistent with the production of a less homogeneous material. Such secondary reactions hinder the reformation of the original urethane network during reprocessing.

Figure 3 presents the overlaid thermograms for each network, its reprocessed counterpart, and their respective first derivatives. Very different behaviour can be observed for the two reprocessed counterparts.

PU1_P exhibits an initial weight loss around 100 °C, likely attributed to moisture absorption during the reprocessing phase rather than structural alterations, as supported by the extra peak in the IR spectra in the hydroxyl region (Figure 2). Although the two-step degradation process is maintained, the first degradation step occurs at lower temperatures (275 °C), while the primary degradation step remains unchanged, with T_5%_ values corresponding to 287 °C. Additionally, the reprocessed material shows a residual mass at 600 °C, corresponding to 6 wt.%, higher than the pristine sample. It is hypothesised that reprocessing affected PU1_P in two ways: reducing the network size, as suggested by the lower thermal stability, and promoting char formation, evident from the increased residual mass. This phenomenon is likely due to stable carbonaceous compounds formed by the coalescence of the hard segment [35].

In Figure 3a, PU2_P exhibits a completely different degradation profile compared with the pristine material. Multiple degradation processes of the pristine materials are substituted with a two-step degradation process. A shift in the T_5%_, T_max,_ and volatilisation interval can be pointed out. Also, the formation of a residue stable at 600 °C and corresponding to 2 wt.% can be observed.

Concerning PU2 and PU2_P, it can be hypothesised that after reprocessing, a completely rearranged network and so a different material with distinct thermal properties.

Regarding the DSC analyses, no significant variations in T_g_ were observed as reported in Figure 3b. Variations can be observed in the thermal capacity of PU2_P with respect to PU2, confirming the structure rearrangement.

Furthermore, to assess how the reprocessing affected the material properties, DMA analyses and tensile tests were performed to compare modulus and mechanical properties (Table 3).

The modulus was determined in the same manner as for the pristine materials, and for comparison, the values at 50 °C were considered, as the obtained curves showed the same stability range. Figure 4a presents the moduli of the materials and their pristine counterparts. It can be observed that only PU1 retains its initial modulus, with a modulus of 2.88 ± 0.26 MPa for PU1_P. In contrast, PU2_P exhibits a modulus of 1.33 ± 0.15 MPa. From these measurements, the crosslink density was calculated to be 358 ± 32 mol/m^3^ and 165 ± 18 mol/m^3^ for PU1_P and PU2_P, respectively, indicating retention of the network structure in PU1 and a significant loosening in PU2 after reprocessing. This trend is further supported by the swelling measurements reported in Table 1: PU1_P displayed values comparable to those of the pristine network, whereas PU2_P showed a marked increase. Structural degradation relative to the pristine material is also evident from the mass loss data, which indicates an increased soluble fraction in PU2_P, attributable to partial breakdown of the system. Figure 4b displays representative stress–strain curves of the series compared with their respective reprocessed samples. The tensile strength values obtained were 1.00 ± 0.08 MPa for PU1_P and 0.36 ± 0.07 MPa for PU2_P. Meanwhile, the elongation at break values were 103 ± 10% for PU1_P and 83 ± 18% for PU2_P.

A recovery index was introduced to compare the properties before and after reprocessing, defined as RI% = (reprocessed property value)/(pristine property value) × 100. This index was calculated for modulus (RIm), elongation at break (RIε), and tensile strength (RIσ). The obtained values for RIm were 95% for PU1 and 58% for PU2. Regarding RIε, the values were 114% for PU1 and 42% for PU2. Finally, for RIσ, the values were 108% for PU1 and 38% for PU2.

A comparative analysis of the mechanical properties and elastic modulus clearly indicates that effective reprocessing was achieved exclusively for PU1, resulting in a material with characteristics close to the pristine polymer. This outcome is further sustained by the preservation of the network structure, as confirmed by FTIR spectroscopy and thermal analysis. It is also worth emphasising that the processing parameters employed for PU1, namely the conditions identified via DMA, were directly applicable, whereas PU2 required extended processing times, as no compact specimen could be obtained under the same conditions.

These findings are consistent with the hypothesis formulated from the KWW fitting analysis. Specifically, it was proposed that bond opening followed by network reformation can occur only in PU1, where two distinct relaxation processes are believed to contribute, enabling the recovery of properties comparable to the pristine polymer. Conversely, PU2 displayed only a single relaxation process; consequently, not only was an extended processing duration necessary to obtain a consolidated specimen, but the resulting material also exhibited a pronounced deterioration in properties. These observations give rise to two key considerations.

The first consideration concerns the methodology for determining reprocessing conditions in networks with low crosslink density, strongly divergent from ideal networks. Although DMA analysis can detect relaxation phenomena and, in line with the classical CAN framework, be employed to define temperature and time parameters, such conditions do not necessarily correspond to effective reprocessing settings. In the case of PU2, this characterisation suggested shorter processing times; however, these conditions proved insufficient. Consistent with the literature, a material with lower crosslink density exhibited faster relaxation, yet this could not be directly associated with network rearrangements as described by CAN theory. The need to extend the processing duration to obtain a consolidated specimen indicates that bond-exchange-driven relaxation occurs at a slower rate than other relaxation processes within the material.

The second consideration relates to crosslink density itself. It has been observed that, as this parameter increases, the recovery of mechanical properties relative to the pristine material becomes more effective. It is hypothesised that crosslink density plays a critical role in restoring both the polymer network and its associated properties. In more densely crosslinked systems, the dynamic bonds responsible for bond scission and reformation are in closer proximity, which facilitates more efficient recombination and yields a reprocessed network similar to the pristine material, as observed for PU1. This interpretation is supported by the fact that, even when extended processing times were applied to PU2 to produce a reprocessed specimen, the resulting material exhibited markedly different properties and a significantly lower recovery capacity compared to PU1.

## 4. Conclusions

Recent advances in CANs have highlighted their potential for reprocessing crosslinked polymers via efficient bond exchange. DCC has recently been extended to systems closer to industrial thermosets, suggesting prospects for scalable recycling strategies. A critical challenge remains understanding property retention after reprocessing, which is essential for rational material design. This work explores the relationship between crosslink density and mechanical performance retention in model polyurethane networks with varying degrees of crosslinking, aiming to clarify its role in reprocessability.

Two different PU networks were synthesised and characterised via FTIR, DSC, TGA, DMA, and tensile testing and subsequently reprocessed via hot-pressing. The reprocessing conditions were determined through stress relaxation and the Maxwellian approach to determine τ*. This approach proved successful only for PU1, whereas PU2 yielded a non-coherent material. Only by extending the reprocessing time was it possible to obtain a homogeneous and characterizable specimen. Interest was given to retention of mechanical properties observed only in PU1 (RIm = 95%, RIε = 114% and RIσ = 108%) while PU2 exhibited markedly lower values (RIm = 58%, RIε = 42% and RIσ = 38%). We suggest that this behaviour arises from the differences in crosslink density and, through the application of the KWW fitting, we could suppose that in the more densely crosslinked network, relaxation can be attributed in part to dynamic covalent exchange, whereas in the looser network, relaxation is primarily influenced by soft segments, preventing effective network reformation.

These findings highlight not only the need to reconsider the use of relaxation analyses as a straightforward indicator of bond-exchange efficiency, but also, and more importantly, the pivotal role of crosslink density in governing the reprocessability of thermoset polyurethanes.

## Figures and Tables

**Table 1:** Summary of thermal values compared between pristine and reprocessed materials.

Sample	T5% (°C)	Tmax (°C)	Tg (°C)	Swelling (%)	Mass Loss (%)
PU1	297	396	−27	196 ± 4	8.3 ± 0.8
PU1_P	287	396	−26	209 ± 15	7.7 ± 0.2
PU2	265	360	−63	242 ± 2	4.4 ± 0.5
PU2_P	280	377	−63	287 ± 3	7.2 ± 0.4

**Table 2:** Summary of the values obtained with the KWW fitting and comparison of the values obtained for relaxation time and activation energy using the KWW method and DMA analysis.

Sample	τ^KWW^	β	R^2^	<τ> (s)	E_a_^KWW^ (KJ/mol)	τ* (s)	E_a_ (KJ/mol)
PU1 150 °C	3141	0.91	0.99	3276	64.6	3379.2	88.5
PU1 160 °C	2467	0.87	0.99	2639		2565	
PU1 170 °C	1347	0.89	0.99	1426		1080	
PU2 150 °C	1693	1	0.97	1693	127.7	1510.2	124.8
PU2 160 °C	939	1	0.98	939		1089.6	
PU2 170 °C	317	1	0.99	317		586.2	

**Table 3:** Summary of mechanical properties and modulus evaluated in pristine and reprocessed materials and associated recovery index.

Sample	Tensile Strength (MPa)	RIσ (%)	Elongation at Break (%)	RIε (%)	Storage Modulus (Mpa)	RIm (%)	υ_e_ (mol/m^3^)
PU1	0.93 ± 0.12		90 ± 16		3.05 ± 0.47		379 ± 58
PU1_P	1.00 ± 0.08	108	103 ± 10	114	2.88 ± 0.26	95	358 ± 32
PU2	0.96 ± 0.14		197 ± 35		2.30 ± 0.27		286 ± 33
PU2_P	0.36 ± 0.07	38	83 ± 18	42	1.33 ± 0.15	58	165 ± 18

## Data Availability

Data are contained within the article.
